# Prevalence and socio-economic distribution of hazardous patterns of alcohol drinking: study of alcohol consumption in men aged 25–54 years in Izhevsk, Russia

**DOI:** 10.1111/j.1360-0443.2006.01693.x

**Published:** 2007-04-01

**Authors:** S Tomkins, L Saburova, N Kiryanov, E Andreev, M McKee, V Shkolnikov, D A Leon

**Affiliations:** 1 London School of Hygiene and Tropical Medicine London, UK; 2 Social Technologies Institute Izhevsk, Russia; 3 Izhevsk Medical Academy Izhevsk, Russia; 4 Research Institute of Statistics Goskomstat, Moscow, Russia; 5 Max Plank Institute Rostock, Germany

**Keywords:** Alcohol, hazardous drinking, premature mortality, Russia, socioeconomic

## Abstract

**Aim:**

To estimate the prevalence of hazardous drinking and its socio-economic distribution among Russian men.

**Design:**

Participants were an age-stratified, population-based random sample of men aged 25–54 years living in Izhevsk, a city in the Urals, Russia. Interviewers administered questionnaires to cohabiting proxy respondents about behavioural indicators of hazardous drinking derived from frequency of hangover, frequency of drinking beverage spirits, episodes in the last year of extended periods of drunkenness during which the participant withdraws from normal life (zapoi), consumption of alcoholic substances not intended to be drunk (surrogates) and socio-economic position. Logistic regression was used to examine associations between socio-economic position and indicators of hazardous drinking in the past year.

**Findings:**

Of 1750 men, 79% drank spirits and 8% drank surrogates at least sometimes in the past year; 25% drank spirits and 4% drank surrogates at least weekly and 10% had had an episode of zapoi in the past year. After adjustment for other socio-economic factors, education was strongly associated with indicators of hazardous drinking. Men with the lowest level of education compared to the highest level of education had an odds ratio of surrogate drinking of 7.7 (95% CI 3.2–18.5), of zapoi of 5.2 (2.3–11.8) and of frequent hangover of 3.7 (1.8–7.4). These indicators of hazardous drinking were also independently strongly associated with being unemployed (versus employed) and with levels of household wealth/amenities. Associations of all these variables with daily consumption of beverage spirits were weaker.

**Conclusion:**

Using a novel range of indicator variables of hazardous drinking, this paper shows that the prevalence of these behaviours is high among working-age men in this Russian city. Moreover, these hazardous behaviours show very clear socio-economic patterns, with particularly high prevalence among those who have had the least education and are not in employment. In contrast, more conventional measures of heavy drinking, based on frequency of consumption of beverage spirits, are less prevalent and show much weaker associations with socio-economic position.

## BACKGROUND

Russia is one of the very few industrialized countries in the world where life expectancy has been declining [1]. In 2003, male life expectancy at birth was 58.8 years, lagging behind EU member states by about 16 years [2]. Alcohol is thought to one of the major proximal determinants of the dramatic fluctuations in mortality observed in Russia since 1985 [2–5] that have been particularly marked among working-age men. For example, there was an increase in life expectancy following the start of the anti-alcohol campaign in 1985 [6] that led to falls in levels of per capita alcohol consumption in Russia [7–9]. Rates of mortality from alcohol-related deaths among working-age men decreased particularly sharply [9]. However, with the collapse of the Soviet Union thisprogress was reversed, with a 6-year decline in male life expectancy between 1990 and 1994 that coincided with increases in per capita alcohol consumption.

Data on alcohol consumption in Russia, particularly for the past decade, are considered unreliable [7], as there is considerable unregistered trade and production [7, 9–11], the bulk of which is thought to have evaded official records [12]. Those estimates that have been produced vary widely: in 1992 the official estimate was 5.0 litres of pure alcohol per capita per annum [13], which is relatively low compared to other countries [7, 12–14]. Indirect attempts to estimate alcohol consumption levels using methods including sugar sales [15] or indicators of alcohol-related harm [12] placed 1992 levels of consumption at 13 litres per capita per annum [12, 15, 16] and overall alcohol consumption level in 1997 at 13–15 litres of pure alcohol per capita [7] and rising [17].

A number of surveys [12, 18–26] have attempted to estimate alcohol consumption levels directly. These have tended to focus on typical alcoholic beverage consumption (beer, wine and spirits). Such surveys tend to yield relatively low per capita consumption figures, which may be explained partly by under-reporting [27]. Nevertheless, some of these surveys have shown that prevalence of ‘binge drinking’ of beverage alcohols is relatively high [24, 28, 29]. However, there are no documented studies that have explicitly collected information on two important aspects of hazardous drinking in Russia: (i) zapoi (periods of continuous drunkenness lasting several days) and (ii) consumption of surrogate alcohols (manufactured substances containing up to 95% ethanol by volume but not officially intended for drinking [30], such as eau de cologne, alcohol-containing medicines, fluids for lighting fires and industrial and technical spirits, including window cleaner). This is a particularly important absence in the literature, as patterns of hazardous drinking such as these, or other indicators of underlying hazardous drinking behaviours such as having frequent hangovers, may related be more strongly to health and mortality than conventional summary measures of average alcohol consumption [24].

The term ‘hazardous drinking’ is being used here to describe a range of extreme alcohol-related behaviours. This paper seeks to investigate the relationship between four such behaviours and selected socio-economic factors. Developing our understanding of the prevalence and socio-economic distribution of these behaviours is a prerequisite for developing contextually appropriate interventions.

## DATA AND METHODS

We have analysed data from a population sample of men aged 25–54 years recruited as controls in a case–control study of premature male mortality conducted in Izhevsk, Russia. This is a typical, medium-sized Russian industrial town, population 650 000, located on the western side of the Ural mountains. The study began in December 2003 following an earlier prototype investigation [31]. Cases were all deaths among male residents of Izhevsk aged 25–54 years occurring over a 24-month period, while controls were selected at random from the official voters list (compiled in 2003). Controls were frequency matched by age to the cases. As most of the information about cases and controls came from interviews with proxy informants living in the same household as the study participants, we excluded any cases or controls who lived alone.

Between 18 December 2003 and 16 November 2005, 3078 potential controls were selected. Members of a team of 25 trained interviewers visited each address. Participant addresses from the electoral roll were verified at the city's address bureau and those found were visited up to three times in order to locate respondents, with the following results: the address did not exist or was not listed for 404 individuals; for 76 men there was no answer at the door; 157 men lived alone and were therefore excluded; in 45 households, no suitable proxy respondent was found or the man had died. Of the remaining 2396 eligible households, 646 proxies refused or were not available. However, 1750 successful proxy interviews were obtained, representing a response rate of 60% of those approached where the proxy was not known to have died or to be living alone. Eighty-five per cent of interviews were with the spouse, 9% with a parent and 2% or less with other proxy types.

Proxies in each household were identified for interview according to a protocol specifying order of preference (spouse, followed by mother, etc.). The questionnaire was developed following a systematic review of the literature on the validity of proxy informants, and employed a five-stage piloting programme and eight iterations in Russian, with back-translations into English to ensure accurate translation.

The study collected information about hazardous drinking over the previous year, in addition to usual questions about frequency and amount of beverage alcohols (beer, wine and spirits). Two questions investigated frequency of consumption of surrogate alcohols: ‘Please indicate how often alcoholic substances not intended to be drunk were usually drunk?’ (every day or more often, nearly every day, three or four times a week, once or twice a week, 1–3 times a month, a few times a year, no answer); ‘On which day of the week were alcoholic substances not intended to be drunk usually drunk?’ (only at the weekend, only on weekdays, any day, every day, only on holidays/celebrations, no answer). Responses to the following questions were also obtained: ‘How often does he have a hangover?’; ‘Has he had one or more episodes of zapoi in the past year/month/week?’. Questions about alcoholic beverage consumption included frequency and usual amount consumed of beer, wine and spirits. Selected socio-economic information was collected, including education level, employment status, household car ownership and whether or not the household had central heating. Hazardous drinking was defined as any of the following in the past year: having drunk surrogates; having been on zapoi; having frequent hangovers (once per month or more); having drunk spirits daily.

In addition to interviewing proxies for controls we also interviewed the controls themselves. However, we have analysed the information obtained from proxy informants, as we believe that this is less affected by the tendency for under-reporting than self-reported alcohol consumption [32]. In our data, 12% of proxies reported that the man had been on zapoi at least once in the past year, compared with only 8% of men self-reporting the same behaviour; 15% of proxies reported that the man had frequent hangovers during the past year, compared with 10% of men self-reporting this. Registration at the city alcohol-treatment clinic, information on which was obtained for all participants, provided a measure free of potential proxy-reporting bias of alcohol problems. The view that proxy information on alcohol is at least as (if not more) reliable as self-reports [33, 34] was supported by an analysis of the strength of association between self- and proxy reports of hazardous drinking with this measure. The association of narcology registration with hazardous drinking was stronger when hazardous drinking exposure was proxy-reported than when it was self-reported (consumed surrogates: OR = 8.6 from proxy-report, 8.0 from self-report; zapoi: OR = 7.9 from proxy-report, 6.5 from self-report).

A selection of variables relating to specific aspects of socio-economic status was selected in order to explore the complex pathways between these and hazardous drinking behaviours. These were education level attained (incomplete secondary; secondary, specialized/professional; incomplete/complete higher), whether the man was in employment (regular paid; unemployed due to invalidity; unemployed due to ill health; unemployed for other reasons) and an amenity index described by presence/absence of household central heating and/or car ownership. For these variables there is ‘almost perfect’ [35] agreement between information provided by men and proxies (kappa = 0.96 for household size, 0.82 for education level, 0.87 for being in employment, 0.83 for car ownership).

Socio-economic conditions may in themselves be causal determinants of hazardous drinking. However, hazardous drinking may also affect socio-economic circumstances. Of the variables we have selected, education is the least likely to be influenced by current or recent drinking habits (although those who began drinking at a young age may have failed to complete their education). However, a low education level may plausibly be a marker or driver of a tendency to drink more hazardously. Car ownership was not particularly common among households in Izhevsk (44%), and is a useful variable to identify households with above-average income. Central heating is usually provided centrally in Russia, a country whose winter temperatures fall well below freezing, and the absence of central heating indicates a very poor housing standard. A combination of these two variables helps to differentiate the socio-economic position of men which may, plausibly, be linked to hazardous drinking behaviours. Finally, being in employment may be a particularly important factor in hazardous drinking: hazardous drinking may lead to job loss, although it is also possible that unemployment may lead to hazardous drinking. This, in turn, may lower the probability of re-employment. These complexities need to borne in mind when interpreting our analyses, although the cross-sectional data we have collected are unable, alone, to disentangle the direction of causality between hazardous drinking and socio-economic characteristics.

### Statistical methods

The prevalence of hazardous drinking in this population was determined. Logistic regression was used to obtain odds ratios for the selected hazardous drinking behaviours with respect to the three socio-economic variables identified above (education, employment status, amenity index), each of which were introduced into the models as categorical variables. The crude effect of each socio-economic factor was obtained for each type of hazardous drinking behaviour, and the effect of adjustment for each and both of the other socio-economic factors was examined in order to evaluate independent effects. Subsets of complete data were used in the examination of the effect of socio-economic factors on each separate type of hazardous drinking behaviour, in order to facilitate legitimate comparison of effects. All associations were adjusted for age. Distribution tables were directly standardized according to the age and marital status of the Izhevsk population (Table 1) derived from the voters list (July 2002) Analyses were conducted using STATA, version 8.2.

**Table 1:** Percentage of male population of Izhevsk, from the electoral roll and of the male study participants, by age group and marital status distribution.

Percentage of distribution: Izhevsk reference population (study population)						
Marital status	25–29 years	30–34 years	35–39 years	40–44 years	45–49 years	50–54 years
Registered marriage	9.3 (3.3)	9.6 (5.6)	10.6 (5.4)	13.6 (13.8)	13.5 (20.7)	12.3 (28.5)
Unregistered marriage	2.1 (1.3)	1.7 (1.1)	1.4 (1.5)	1.4 (1.1)	1.1 (1.9)	0.8 (3.0)
Never married	6.3 (0.7)	2.5 (0.4)	1.4 (0.6)	1.2 (1.1)	0.8 (1.3)	0.5 (2.1)
Widowed	0.0 (0.0)	0.0 (0.0)	0.1 (0.0)	0.2 (0.2)	0.3 (0.2)	0.4 (0.5)
Divorced	1.0 (2.3)	1.4 (1.1)	1.6 (0.6)	2.0 (0.6)	1.7 (0.7)	1.4 (0.6)

## RESULTS

Table 2 shows the age-standardized distribution of men by frequency of drinking different types of alcohol. Approximately 80% of men drank beer at least once per month; approximately one-tenth drank beer daily. Wine was a less popular beverage, with more than half thepopulation never drinking it; approximately only 7% of those who did drink wine did so weekly or more often. In contrast, more than 80% drank spirits, approximately a quarter drinking spirits weekly or more often and approximately 3% drinking spirits daily. Consumption of surrogates was less frequent than consumption of beer, wine or spirits. Table 3 shows the distribution of hazardous drinking behaviours. Daily/almost daily spirit consumption was the least frequent of the four measures examined. Less than 1% (15) had displayed all four hazardous drinking behaviours in the past year. Approximately 4% displayed two or three, while 12% displayed only one and the remaining 79% displayed none of these hazardous drinking behaviours. There is substantial overlap between men who drink surrogates and those who go on zapoi. Of the 135 men reported to have drunk surrogates in the past year, 64% had been on zapoi. Of 183 men who had been on zapoi, 48% had drunk surrogates.

**Table 2:** Age- and marital-status standardized distribution of study population by frequency of consumption of different types of alcoholic drink.

Frequency of consumption	Beer %*	(n)	Wine %*	(n)	Spirits %*	(n)	Surrogates %*	(n)
Daily	8.6	(145)	1.4	(25)	3.2	(63)	2.0	(41)
Weekly	40.1	(658)	5.3	(103)	20.7	(427)	2.1	(42)
Monthly or less	29.7	(513)	32.8	(542)	53.8	(887)	3.0	(52)
Never/almost never	20.2	(415)	58.4	(1060)	21.0	(355)	90.9	(1590)
No response	1.3	(19)	2.1	(20)	1.3	(18)	1.9	(25)
Total	100.0	(1750)	100.0	(1750)	100.0	(1750)	100.0	(1750)

* Due to direct standardization to the Izhevsk city male population 2003, the percentages above do not correspond to the crude percentages which can be derived from the sample distribution presented here.

**Table 3:** Age- and marital-status standardized distribution of hazardous drinking behaviours in the past year.

	Age standardized	
	
Question	%*	(n)
Consumed surrogates?		
Yes	7.2	(*135*)
No	90.9	(1590)
No response	1.9	(25)
Total	100.0	(1750)
Went on zapoi?		
Yes	10.6	(*183*)
No	78.1	(1348)
No response	11.2	(219)
Total	100.0	(1750)
Frequent hangovers?		
Yes	12.8	(*224*)
No	73.0	(1260)
No response	14.2	(266)
Total	100.0	(1750)
Spirits consumption frequency		
Daily or almost daily	21.0	(*63*)
Less often or not at all	77.7	(1669)
No response	1.3	(18)
Total	100.0	(1750)

* Due to direct standardization to the Izhevsk city male population 2003, the percentages above do not correspond to the crude percentages which can be derived from the sample distribution presented here.

Table 4 shows the prevalence of hazardous drinking behaviours by socio-economic factors. The strength of association of each socio-economic factor with hazardous drinking estimated using logistic regression is shown in Tables 5, 6 and 7. In each table, odds ratios for each type of hazardous drinking in relation to a specific socio-economic factor are shown with and without adjustment for each of the other two socio-economic factors. In general, those with lower education, unemployment and not having a car or central heating were most likely to have proxy reports of these hazardous drinking behaviours. However, daily/almost daily consumption of spirits was associated less consistently and less strongly with socio-economic position than the other markers of hazardous drinking. Overall, the associations of hazardous drinking with employment status changed little on adjustment for education and household amenities. In contrast, the associations with education and amenities were attenuated on adjustment although, with the exception of daily/almost daily spirits drinking, there was clear evidence from the fully adjusted models that each had an independent effect. Of surrogates, zapoi and frequent hangover, the latter showed the weakest associations with each of the socio-economic measures.

**Table 4:** Age standardized distribution of selected hazardous drinking behaviours in the past year by socio-economic factors.

	Consumed surrogates % (n/total)	Went on zapoi % (n/total)	Had frequent hangovers % (n/total)	Drank spirits daily % (n/total)
Education level				
Incomplete secondary or less	25.5 (22/97)	24.8 (21/81)	41.7 (21/79)	4.5 (5/98)
Secondary, specialized or professional	8.0 (103/1219)	14.4 (146/1076)	17.3 (178/1039)	3.7 (45/1226)
Incomplete/complete higher	1.6 (9/395)	2.7 (14/365)	5.2 (22/357)	2.1 (13/394)
In employment?				
Regular paid employment	3.7 (70/1432)	6.3 (93/1267)	10.8 (142/1228)	2.2 (41/1436)
Unemployed, other reasons	26.2 (59/197)	48.8 (81/190)	35.3 (67/185)	10.3 (20/198)
Unemployed, invalidity	1.5 (3/78)	2.3 (4/57)	25.2 (9/55)	0.7 (2/79)
Unemployed, ill health	4.9 (3/16)	34.4 (5/15)	34.4 (5/14)	0.0 (0/17)
Amenity index				
Neither car nor central heating	22.2 (24/124)	33.9 (27/108)	34.3 (27/105)	1.1 (4/124)
Either car or central heating	8.4 (89/936)	12.3 (110/841)	16.6 (140/808)	4.0 (42/942)
Both car and central heating	3.4 (22/665)	8.3 (46/582)	9.2 (57/571)	1.6 (17/666)

Due to direct standardization to the Izhevsk city male population 2003, the percentages above do not correspond to the crude percentages which can be derived from the sample distribution presented here.

**Table 5:** Association between education level and hazardous drinking behaviours in the past year, adjusted for other socio-economic factors.

	*Incomplete secondary or less*		*Secondary, specialised or professional*		*Incomplete/complete higher*	
				
	*OR*	*(95% CI)*	*OR*	*(95% CI)*	*OR (95% CI)*	P for trend*
Ever consumed surrogates						
Unadjusted	12.8	(5.6, 29.2)	3.9	(1.9, 7.7)		< 0.001
Adj. for employment status	9.6	(4.0, 22.8)	3.4	(1.7, 6.9)	1.0 (referent)	< 0.001
Adj. for amenity index	8.8	(3.8, 20.5)	3.4	(1.7, 6.9)		< 0.001
Adj. for both	7.7	(3.2, 18.5)	3.2	(1.6, 6.5)		< 0.001
Had been on zapoi						
Unadjusted	9.4	(4.5, 19.7)	4.0	(2.3, 7.0)		< 0.001
Adj. for employment status	6.0	(2.7, 13.3)	3.3	(1.8, 5.8)	1.0 (referent)	< 0.001
Adj. for amenity index	7.2	(3.4, 15.4)	3.7	(2.1, 6.5)		< 0.001
Adj. for both	5.2	(2.3, 11.8)	3.2	(1.8, 5.7)		< 0.001
Had a hangover frequently						
Unadjusted	5.8	(3.0, 11.3)	3.1	(2.0, 5.0)		< 0.001
Adj. for employment status	4.2	(2.1, 8.5)	2.7	(1.7, 4.4)	1.0 (referent)	< 0.001
Adj. for amenity index	4.6	(2.3, 9.1)	2.9	(1.8, 4.6)		< 0.001
Adj. for both	3.7	(1.8, 7.4)	2.6	(1.6, 4.2)		< 0.001
Drank spirits daily versus less frequently						
Unadjusted	1.6	(0.6, 4.7)	1.1	(0.6, 2.0)		0.51
Adj. for employment status	1.3	(0.4, 3.9)	1.0	(0.5, 1.8)	1.0 (referent)	0.82
Adj. for amenity index	1.4	(0.5, 4.2)	1.0	(0.5, 1.9)		0.71
Adj. for both	1.3	(0.4, 3.8)	0.9	(0.5, 1.7)		0.91

* *P*-value for χ^2^ test for a general association (non-ordinal variables).

All analyses are adjusted for age group.

**Table 6:** Association between employment status and hazardous drinking behaviours, adjusted for other socio-economic factors.

	*Regular paid employment*	*Unemployed, other reasons*		*Unemployed, invalidity*		*Unemployed, ill health*		P for association*
					
	*OR (95% CI)*	*OR*	*OR (95% CI)*	*OR*	*(95% CI)*	*OR*	*(95% CI)*	P for association*
Ever consumed surrogates								
Unadjusted		8.8	(5.9, 13.1)	0.8	(0.2, 2.5)	4.8	(1.3, 17.3)	< 0.001
Adj. for employment status	1.0 (referent)	8.0	(5.3, 11.9)	0.6	(0.2, 2.1)	2.8	(0.7, 10.6)	< 0.001
Adj. for level of education		7.5	(5.0, 11.2)	0.6	(0.2, 2.0)	4.0	(1.1, 15.1)	< 0.001
Adj. for both		7.1	(4.7, 10.7)	0.5	(0.2, 1.8)	2.7	(0.7, 10.5)	< 0.001
Had been on zapoi								
Unadjusted		9.8	(6.8, 14.1)	1.0	(0.3, 2.7)	6.6	(2.2, 20.2)	< 0.001
Adj. for employment status	1.0 (referent)	8.8	(6.0, 12.7)	0.9	(0.3, 2.4)	4.5	(1.4, 14.0)	< 0.001
Adj. for level of education		8.8	(6.1, 12.8)	0.9	(0.3, 2.5)	5.7	(1.8, 17.5)	< 0.001
Adj. for both		8.2	(5.6, 11.9)	0.8	(0.3, 2.3)	4.1	(1.3, 12.9)	< 0.001
Had a hangover frequently								
Unadjusted		4.4	(3.1, 6.3)	1.6	(0.7, 3.3)	4.2	(1.4, 12.9)	< 0.001
Adj. for employment status	1.0 (referent)	3.9	(2.8, 5.6)	1.4	(0.7, 2.9)	3.0	(1.0, 9.2)	< 0.001
Adj. for level of education		4.0	(2.8, 5.7)	1.4	(0.7, 2.9)	3.6	(1.2, 11.2)	< 0.001
Adj. for both		3.7	(2.5, 5.3)	1.3	(0.6, 2.7)	2.8	(0.9, 8.7)	< 0.001
Drank spirits daily versus less frequently								
Unadjusted		4.1	(2.3, 7.2)	0.9	(0.2, 3.7)	–	–	< 0.001
Adj. for employment status	1.0 (referent)	4.1	(2.3, 7.2)	0.9	(0.2, 3.6)	–	–	< 0.001
Adj. for level of education		4.0	(2.2, 7.1)	0.8	(0.2, 3.4)	–	–	0.001
Adj. for both		4.0	(2.2, 7.2)	0.8	(0.2, 3.4)	–	–	0.001

* *P*-value for χ^2^ test of no association, on 1 degree of freedom.

All analyses are adjusted for age group.

**Table 7:** Association between amenity index and hazardous drinking behaviours, adjusted for other socio-economic factors.

	*Neither car nor central heating*		*Either car or central heating*		*Both car and central heating*	
				
	*OR*	*(95% CI)*	*OR*	*(95% CI)*	*OR (95% CI)*	P for trend*
Ever consumed surrogates						
Unadjusted	7.3	(3.9, 13.6)	3.1	(5.1, 2.2)		< 0.001
Adj. for employment status	4.5	(2.3, 8.8)	2.7	(4.4, 2.2)	1.0 (referent)	< 0.001
Adj. for level of education	5.4	(2.9, 10.3)	2.7	(4.4, 2.2)		< 0.001
Adj. for both	3.6	(1.8, 7.1)	2.4	(4.0, 2.2)		< 0.001
Had been on zapoi						
Unadjusted	4.2	(2.4, 7.2)	1.8	(2.6, 2.2)		< 0.001
Adj. for employment status	2.4	(1.3, 4.3)	1.5	(2.2, 2.2)	1.0 (referent)	< 0.001
Adj. for level of education	3.2	(1.9, 5.6)	1.6	(2.3, 2.2)		< 0.001
Adj. for both	2.0	(1.1, 3.7)	1.3	(2.0, 2.2)		0.03
Had a hangover frequently						
Unadjusted	3.2	(1.9, 5.4)	1.9	(2.6, 2.2)		< 0.001
Adj. for employment status	2.2	(1.3, 3.8)	1.7	(2.3, 2.2)	1.0 (referent)	< 0.001
Adj. for level of education	2.6	(1.5, 4.3)	1.7	(2.3, 2.2)		< 0.001
Adj. for both	1.9	(1.1, 3.2)	1.5	(2.1, 2.2)		0.01
Drank spirits daily versus less frequently						
Unadjusted	1.3	(0.4, 3.9)	1.9	(1.1, 3.3)		0.12
Adj. for employment status	0.9	(0.3, 2.7)	1.6	(0.9, 3.0)	1.0 (referent)	0.48
Adj. for level of education	1.2	(0.4, 3.8)	1.8	(1.0, 3.3)		0.15
Adj. for both	0.8	(0.3, 2.6)	1.6	(0.9, 3.0)		0.52

* *P*-value for χ^2^ test for a general association (non-ordinal variables).

All analyses are adjusted for age group.

The analyses described above were replicated using self-reported data, in order to confirm that the use of proxy data is justified. Overall associations between exposures and drinking behaviours were similar, although strength sometimes differed.

## DISCUSSION

This study provides the first assessment of the prevalence of a range of indicators of hazardous drinking in working-age men and their relationship to socio-economic factors in a Russian city at the present time.

More than 21% of men display behaviours associated with hazardous drinking. Russia has a traditional alcohol profile, with wine consumption not particularly widespread. Conversely, frequent beer consumption is widespread, consistent with evidence of the growth in beer consumption in Russia [36], encouraged until recently by the semi-official view that it should not be considered an alcoholic beverage [37]. Of particular concern is the almost ubiquitous frequent consumption of spirits. The surprisingly high prevalence of zapoi (an episode of continuous drunkenness lasting 2 or more days when the person is withdrawn from normal social life) indicates that this, too, is a significant problem on a large scale. Equally alarmingly, our study suggests that 7% of working-age men consume surrogate alcohols. Despite numerous anecdotal reports of surrogate consumption, especially following the 1985 anti-alcohol campaign [6], there is almost nothing in the international literature investigating this dangerous activity; this is, to our knowledge, the first systematic study of surrogate use in a population-based sample in Russia. It is known, however, that surrogate drinking as we have observed it is not a peculiarity of present Russian life. Surrogate alcohols have been a major public health issue in Finland at various points in the past 50 years [38], and eau de cologne drinking was established in Russia during the 1920s [39].

What are the pathways by which these socio-economic factors influence hazardous drinking behaviours? Education level as a determinant of all hazardous drinking is not unanticipated: it is well documented that this component of socio-economic status is related inversely to alcohol consumption levels [17]. More striking is the association among those reporting unemployment for reasons unrelated to health. A similar finding was reported by a Scottish study [40], which found that drinkers who were unemployed self-reported higher alcohol consumption, and a greater percentage of the unemployed reported binge-drinking. These findings indicate that lack of economic and/or social stability may be a key factor driving these behaviours, especially surrogate consumption [41, 42]. Associations observed with the amenity index support these conclusions further. Surrogate consumption may be the end-point of a downward economic spiral: men struggling economically are driven to alcoholism and eventually begin consuming surrogates. The combination of lower price and higher alcohol concentration means that a given quantity of alcohol from surrogates is typically about one-sixth the price of alcohol from vodka [30]. It seems plausible that removal of a price barrier leads to a vicious downward spiral, with progressive withdrawal from society. The wide availability and cheap cost of surrogates are two characteristics of these substances that should be tackled in interventions. Although the magnitude of effects for zapoi are lower than those associated with surrogate consumption, this type of hazardous drinking is more widespread and hence may have greater absolute consequences for population health.

The inconsistent pattern of the socio-economic determinants of daily spirits consumption may be explained partly by the possibility that those who can afford to drink spirits every day are unlikely to be in the lowest socio-economic groups. Although, overall, drinking spirits is widespread compared with the other three hazardous drinking behaviours, the small group of men who drink spirits daily are perhaps in an unsustainable situation, on the brink of entering, rather than already caught within, a downward socio-economic spiral which would explain the lack of an association with the amenity index. Indeed, it appears that those who can afford to do so choose to drink spirits. It is the least fortunate in society who are forced to turn to surrogate alcohols. The absence of an effect of the lowest level of education on spirits consumption may be explained partly by those with low education being less likely to drink spirits regularly, due to economic obstacles. Furthermore, among the small number of men reported to be unemployed due to ill health, none drank spirits daily. This suggests that those men who had become unemployed as a consequence of the health effects of alcoholism were no longer able to afford to drink spirits.

**Figure 1:**
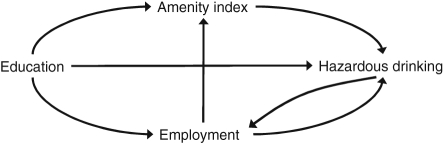
Relationships between socio-economic factors and hazardous drinking behaviours

One possible way of looking at the inter-relationship of the various socio-economic factors with hazardous drinking that we have considered is presented schematically in Fig. 1. As shown, there may be circularity in associations between socio-economic factors and hazardous drinking behaviours. The education level attained measures events in the past, and is therefore the least likely of the factors examined here to be affected by currenthazardous drinking behaviour or current socio-economic status, although it is conceivable that these variables are influenced by vertical pathways which influence one another across generations. The group categorized as unemployed due to ill health is likely to contain men who stopped work due to genuine ill health, who may therefore be less likely to drink. It is hypothesized that this group also includes men who became unemployed due to alcoholism or associated health problems, and their categorization as ‘unwell’ is a euphemism for this. The existence of this reverse causality has been identified elsewhere [41, 43, 44]. Unemployment and its association with hazardous drinking is undoubtedly a complex and difficult issue, but should not be overlooked as an integral part of the alcohol problem in Russia. A recent analysis using successive waves of the Russian Longitudinal Monitoring Survey has confirmed that alcohol consumption is associated significantly with being fired within the following year [45].

These data have a number of limitations. We did not collect information on biomarkers of hazardous drinking, on consumption of *samogon* (home-brew) or surrogate alcohols beyond consumption frequency, all of which would have been useful in developing a clearer understanding of drinking behaviours. These data are based on men living with at least one other person, so findings are not generalizable to the whole population. Nevertheless, hazardous drinking prevalence is probably higher among those living alone, so effect measures err on the side of underestimation. Our use of proxies is unusual. However, as we have discussed above, we believe that proxy-reported data provide a picture of associations between socio-economic exposures and indicators of hazardous drinking at least as good as, if not better than, that obtained from self-reports.

In summary, this paper provides the first in-depth analysis of hazardous drinking behaviours and their socio-economic correlates among working-age Russian men, going beyond earlier surveys of frequency and amount of beverage alcohol consumption. This shows a much higher prevalence, and a far stronger relationship with socio-economic factors, of surrogate drinking, zapoi and frequent hangover than with daily or almost/daily consumption of spirits. This suggests that conventional approaches to assessing hazardous drinking, at least in the Russian context, should not be restricted to collecting information on the frequency and amount of consumption of beer, wine and spirits. The strong and robust association of employment status with hazardous drinking underlines that alcohol is not only a major public health issue but is likely to have a major negative effect on the development of the Russian economy.
